# Diagnosis-Related Group-Based Financing of Gynecologic Oncology Clinics: A Systematic Review

**DOI:** 10.3390/healthcare13040349

**Published:** 2025-02-07

**Authors:** Alexandra Titopoulou, Eleftherios Vavoulidis, Chrysoula Margioula-Siarkou, Georgia Margioula-Siarkou, Aristarchos Almperis, Stamatios Petousis, Georgios Mavromatidis, Theodoros Dardavesis, Konstantinos Dinas

**Affiliations:** 1 Hippokration General Hospital of Thessaloniki, 54642 Thessaloniki, Greece; alexandra.titopoulou@gmail.com; 2 Second Department of Obstetrics and Gynecology, School of Medicine, Faculty of Health Sciences, Aristotle University of Thessaloniki, 54124 Thessaloniki, Greece; margioulasiarkouc@gmail.com (C.M.-S.); gmargioulasiarkou@gmail.com (G.M.-S.); arisal1998@gmail.com (A.A.); petousisstamatios@gmail.com (S.P.); mavromag@auth.gr (G.M.); konstantinosdinas@hotmail.com (K.D.); 3 Laboratory of Hygiene, Social & Preventive Medicine and Medical Statistics, School of Medicine, Faculty of Health Sciences, Aristotle University of Thessaloniki, 54124 Thessaloniki, Greece; dardaves@auth.gr

**Keywords:** diagnosis-related group (DRG)-based financing, case-based payment (CBP) system, hospital payment reform, reimbursement, healthcare providers, equity of healthcare, healthcare services efficiency, gynecological clinics

## Abstract

**Background/Objectives**: Diagnosis-related group (DRG)-based financing, a subcategory of case-based payment models, has been established as the primary reimbursement scheme in most high-income countries. Almost 40 years thereafter, the impact of the reform on gynecologic oncology funding and recompensation has not been clearly elucidated. This systematic review aims to address the scarce bibliographic data, consolidate the information regarding DRG-based systems in gynecologic oncology, evaluate the advantages and challenges of its implementation worldwide, and examine alternative and complementary recompensation schemes in this context. **Methods**: A stringent and comprehensive literature review of original articles using online databases (Google Scholar and Pubmed) yielded 173 potential results. **Results**: These were further assessed for relevance and eligibility and, finally, an in-depth investigation of 15 articles was conducted. We concluded that, despite the fact that DRG-based financing may be beneficial in most healthcare scenarios, the reimbursement scheme is challenged in the context of gynecologic oncology. **Conclusions**: An innovative approach comprising a combination of the values of case-based and value-based payment models would extend healthcare services beyond acute treatments and propose new perspectives in holistic healthcare provision in a financially sustainable environment.

## 1. Introduction

Hospitals lie in the epicenter of healthcare provision, with their expenditure surpassing 40% of total healthcare costs. It comes as no surprise that hospital financing is a main focus for policy reform, as well as a recurring target for policymakers. It is empirically proven that restructuring the way that healthcare providers are reimbursed significantly affects the quality and quantity of the services they provide [1]. However, the exact ways that different hospital payment schemes alter healthcare provision and impact accessibility are yet to be elucidated.

The retrospective reimbursement systems that were established prior to the 1980s in the United States were deemed insufficient and raised mounting concerns with regard to the sustainability of American government insurance programs (Medicare, Medicaid, Children’s Health Insurance Program (CHIP), etc.). Hence, to control the volume-driven approach of hospitals and promote outcome-oriented, efficient case-expenditure management, a case-based payment (CBP) system was proposed and was gradually incorporated as the primary source of healthcare funding. The newly proposed funding scheme shifted the focus from independent to holistic healthcare provision. Linking the entire patient’s condition to a fixed prospective payment forces hospitals to form new, efficient, cost-effective strategies of treating patients, relieving the government of insufferable ever-increasing healthcare costs.

Diagnosis-related group-based financing (DRG) was invented at Yale University (USA) and is the most widely established example of the CBP system. DRGs are a patient classification system that standardizes provider reimbursement. DRGs group patient cases into economically homogenous groups, based on various factors, namely diagnosis, comorbidities, treatments, and demographic details, and ensure that providers treat clinically similar patients with the same diagnoses and that procedures are reimbursed at a consistent rate. The amount of reimbursement is predetermined and, in most cases, independent from the individual services provided [2].

DRG financing (DRGF) was initially implemented in some of New Jersey’s hospitals as an experimental effort to contain the cost of healthcare for inpatients and facilitate healthcare transparency in the late 1970s and early 1980s. The success of the scheme led to the prominence of this financial system and its integration as the protagonist in healthcare funding in the US, even to this day. In the following decades, this intervention from healthcare insurance bodies, either public or private, gradually became the cornerstone for hospital payment schemes worldwide.

Since their inception, diagnosis-related groups (DRGs) have been closely tied to the organization and reimbursement of healthcare services, with their application evolving to suit various contexts and healthcare settings. DRGs were specifically developed to streamline the delivery of healthcare services within hospitals, focusing on patients requiring acute care, defined as immediate and specialized treatment for time-sensitive medical needs. These services include the management of severe injuries or illnesses, the stabilization and treatment of urgent medical conditions, and the provision of post-operative care to support recovery from surgeries. By categorizing patients into clinically and economically similar groups, DRGs aim to align hospital reimbursement with the resources required for these high-intensity, short-term treatments. This approach not only ensures fair compensation but also incentivizes hospitals to use resources efficiently, reducing unnecessary procedures and improving overall cost-effectiveness [3].

While DRGs were initially intended for acute inpatient services, adaptations have been applied in post-acute care settings, such as rehabilitation and skilled nursing facilities. These settings often use DRG-like groupings to predict costs and allocate resources. A great example of this is Australia’s version of DRGs, known as Australian refined diagnosis-related groups (AR-DRGs), which incorporate mechanisms to adjust payments for post-acute services in certain contexts. DRGs have occasionally been explored for broader population-level reimbursement models, as well. For instance, pilot programs in some healthcare systems aim to integrate DRG-based payments with population health initiatives to reduce costs across entire patient populations. In Germany, adaptations of DRG-based systems are being tested for use in payment models covering outpatient services or cross-sector care episodes, albeit with significant modifications to the original framework [4].

In addition, countries adopting DRGs have tailored the system to meet their unique healthcare needs, often expanding its scope. For example, in France, the DRG-based Tarification à l’Activité (T2A) system includes payment adjustments for teaching hospitals and hospitals treating complex cases. Similarly, Germany’s DRG system (G-DRGs) incorporates psychiatric and psychotherapeutic services [4]. Last but not least, DRG-based schemes have been utilized for comparative health analysis. The Organisation for Economic Co-operation and Development (OECD) has employed DRG data to compare hospital efficiency and spending across member nations, highlighting variations in healthcare delivery.

The overall cost of cancer care in the USA continues to rise, even after surpassing 200 billion dollars per year in 2020 [5]. According to the Global Cancer Observatory, the age-standardized incidence rate for ovarian cancer in Greece is 4.6 per 100,000 women, with a mortality rate of 2.5 per 100,000 women. The Greek incidence rate, being about 1/10th of the American rate, may reflect differences in healthcare access, diagnostic practices, and population demographics. Greece’s healthcare system, which is more publicly funded and centrally controlled, may result in fewer screenings and a different approach to early detection compared to the U.S., where private healthcare and greater access to advanced diagnostic technologies can lead to higher reported rates. Additionally, factors such as a younger average age at diagnosis and a lower prevalence of risk factors like obesity and hormonal treatments in Greece could further contribute to the disparity in ovarian cancer incidence between the two countries.

Endometrial cancer, while less common in Greece compared to other countries, still poses a notable health challenge and a challenge to the Greek healthcare reimbursement system. Cervical cancer remains a concern, with the World Health Organization reporting a cumulative risk of cervical cancer for women aged 0–74 in Greece [6]

Gynecologic oncology clinics provide a variety of highly specialized services. DRG-based financing seems ideal to standardize the expenses of such a costly sector and promote accessibility and the efficient care of women in need [7]. Better resource management while delivering comprehensive treatments equally benefits patients and providers. The implementation of this system is, however, not equally easily plausible in this case [8]. Gynecologic oncology clinics face a variety of different cases that usually demand high specialization and extensive care. Patients with advanced stage gynecologic cancers require multiple treatments along the course of the disease and usually experience complications. The treatment of these complications is also assigned, in most inpatient cases when an outlier reimbursement is not applicable, to the umbrella of the initial DRG. Therefore, healthcare establishments may lower the quality of the treatment, so as to remain within the DRG budget, or they may shoulder the additional financial burden themselves. Appropriate supportive policies should be implemented to secure the beneficial development of DRG reform. A more flexible reimbursement system with the aim of addressing the complexities of these cases seems imperative [9,10].

Data relating to DRGF in gynecologic oncology clinics are scarce in the literature. The objective of this systematic review is to consolidate the data regarding DRG financing (DRGF) and, from those data, to make deductions regarding gynecologic oncology clinics, while providing a comprehensive overview of the advantages and challenges of this reimbursement scheme in the particular context.

## 2. Materials and Methods

Study design: Employing a systematic and exhaustive methodology, this study aimed to evaluate how different healthcare reimbursement schemes, mainly DRGs, impact healthcare services and to evaluate how advantageous or challenging the implementation of these schemes is, in the context of gynecologic oncology clinics. A thorough exploration of the literature was conducted to locate relevant studies, followed by a stringent selection process based on predetermined inclusion and exclusion criteria.

Literature search: The databases Google Scholar and PubMed were searched to identify relevant studies. Google Scholar aggregates a vast range of academic materials, from peer-reviewed articles to gray literature. Its broad scope ensures that the multidisciplinary aspects of healthcare financing—economics, policies, and medical advancements—are met. PubMed is targeted specifically to biomedical research. Its strict indexing criteria ensure the credibility and the relevance of the information to the topic. The comprehensiveness of Google Scholar and the specialization of PubMed guarantee the credibility and robustness of the search. We included search terms for diagnosis-related group-based financing, case-based payment system, reimbursement, financing, funding, gynecologic clinics, obstetrics, gynecology, and oncology. The Boolean operators “OR” and “AND” were used to combine the search terms, i.e., “diagnosis-related group-based financing” OR “DRG” AND “gynecologic clinics” OR “obstetrics” AND “oncology”. The search algorithm maintained a common structure across databases; however, adjustments were made to ensure optimal search results. Supplementarily, we proceeded to hand-search the reference lists, reviews, and gray literature for relevant articles that the search methodology did not identify.

Study selection and eligibility: This review incorporated controlled before–after studies (CBAs), non-randomized clinical trials (NRCTs), randomized clinical trials (RCTs), interrupted time series studies (ITS), and comparative, retrospective, and observational studies. It also incorporated uncontrolled before–after studies (BAs), even though these are discouraged in Cochrane Effective Practice and Organisation of Care (EPOC) guidelines, to address the issue of scarce bibliography data. DRGs were studied at regional, institutional, and independent levels in all settings of care (primary, secondary, and tertiary). There was no discrimination during selection regarding the above criteria.

Inclusion and exclusion criteria: The study included data regarding high-income countries (HICs), as well as low- and middle-income countries (LMICs). The prerequisite for inclusion was that the study stated outcomes of health expenditure, efficiency, equity of healthcare, and the quality and quantity of services. Inpatient and outpatient care services were both included. All articles in this field published until October 2024 were assessed for eligibility. The studies were sourced up to October 2024. We omitted studies that did not include oncology clinics, as well as review articles, meta-analyses, and studies not published in English. As DRG financing is predominantly a Western concept, though it is now emerging in low- and middle-income countries, any significant foreign articles on the subject have likely been translated. Due to the scarce literature available regarding gynecologic oncology clinics, certain data were retrieved from studies regarding oncology clinics or hospitals and gynecologic clinics that did not only specialize in cancer treatment.

This review was performed in accordance with the PRISMA 2020 (Preferred Reporting Items for Systematic Reviews and Meta-Analyses) guidelines. After the initial literature search, titles and abstracts only were screened by two independent authors for relevance. Disagreements were resolved through consensus or by discussion with a third author. Articles deemed irrelevant were excluded. The retrieved records underwent semi-automatic deduplication using Rayyan, an online tool that streamlines the systematic review process, including the deduplication of studies, efficient screening, and organization through features like blinding, tagging, and advanced filtering [11]. Full-text copies of the remaining articles were assessed for eligibility, as per the Population, Intervention, Comparison, Outcomes and Study (PICOS) criteria, by two blinded reviewers. Inconsistencies were, once again, resolved by consensus or by a third reviewer. The references of the full-text copies were assessed to prevent the potential loss of eligible studies that were missed by the database search (using the snowball procedure). Any discrepancies between reviewers were resolved through discussion with a third reviewer, resulting in no relevant studies being excluded.

Data extraction: Data relevant to the topic of the systematic review were extracted from each article, as well as general information. The following data were extracted from the eligible studies: author, year of publication, country, study design, study population/sample size, information about the control group, and whether the outcomes from the implementation of the DRG reimbursement scheme were positive, negative, or neutral (depicted as +, − or 0). Positive outcomes were defined as reduced hospital stays, cost efficiency, improved patient outcomes, adherence to clinical guidelines, financial sustainability, and equitable access to healthcare services. The relevant data were extracted and presented in the format of a table. All records were managed in an Excel spreadsheet.

Quality assessment: We conducted a quality assessment of the included studies using the Newcastle–Ottawa Scale (NOS). This tool evaluates selection, comparability, and outcome/exposure domains, with a maximum score of 9 stars indicating high methodological quality.

## 3. Results

### 3.1. The Selection Process for Included Studies

**Figure 1:**
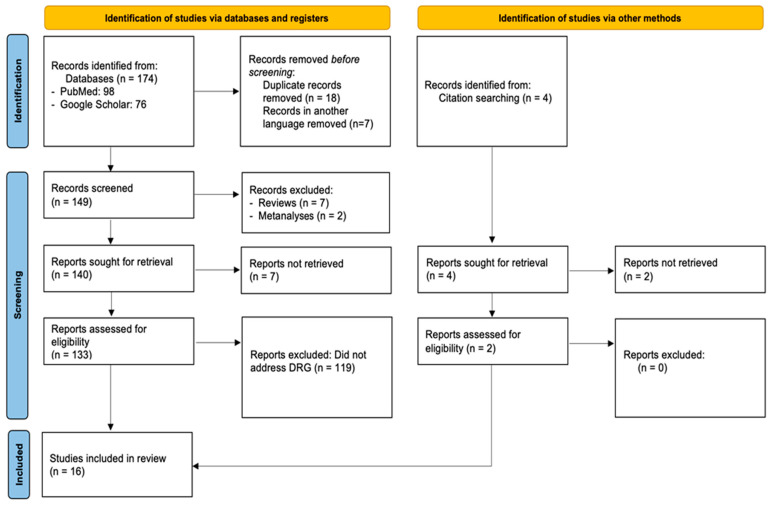
Flow diagram of the selection process of the included studies.

A flow diagram of the selection process, including the reasons for the exclusion of studies, is presented in Figure 1. In total, 174 papers were identified using the initial search strategy (98 from PubMed and 76 from Google Scholar), of which 18 were removed because they were duplicates and another 7 were excluded because they were not in English. Hence, 149 were considered eligible for title–abstract screening. Upon initial review of the title and/or abstract, 119 articles did not address DRG-based funding of gynecologic oncology clinics, 7 were reviews, and 2 were meta-analyses, while 7 studies were not retrieved. Subsequently, 14 articles were selected for full-text screening, all of which met the inclusion criteria and were included in this review. Of the selected articles, nine were sourced from Google Scholar and five from PubMed. Furthermore, the references of the included studies and references from other relevant studies from high-impact journals were hand-searched, and two papers that were not found in the initial literature search were included as well. Thus, the 16 studies that were included in the final sample investigated in depth the impact of the implementation of DRGF in gynecologic oncology clinics. The characteristics of the included studies can be found in Table 1.

The quality assessment revealed that most studies were of moderate to high quality, with scores ranging from five to nine stars. Studies with high scores were characterized by strong study designs, robust comparisons, and clear outcome reporting. Lower scores were attributed to retrospective designs, limited control of confounding variables, and in-sufficient methodological details. A summary of the scores is provided in Table 2.

### 3.2. Structure of Diagnosis-Related Group Financing

The CBP system follows a very specific structure. The highest level of hierarchy comprises the major diagnostic categories (MDCs). MDCs are large categories that group patient cases; these are not based on specific diagnoses but are based on the system/organ affected or based on the major disease group involved (principal diagnosis). In every country’s DRG system there might be differences in and adaptations to the MDCs. However, the MDCs utilized by centers in the USA provide a widely recognized framework. There are 25 different MDCs, according to Centers for Medicare & Medicaid Services (CMS), the US federal agency that provides health coverage to more than 160 million Americans through Medicare, Medicaid, the Children’s Health Insurance Program, and the Health Insurance Marketplace. The MDCs relating to gynecological oncology are as follows: (i) MDC 12: diseases and disorders of the genitourinary system (e.g., kidney stones, unitary tract infections (UTI) infections, and prostate cancer), (ii) MDC 13: diseases of the female reproductive system (e.g., normal childbirth, cesarean delivery, and complications of pregnancy), and (iii) MDC 16: neoplasms (e.g., various cancers, benign tumors, and metastatic cancers).

Given the above classification, gynecologic oncology cases are usually categorized within MDC 16 (neoplasms) and MDC 12 (diseases and disorders of the genitourinary system), but mostly within MDC 13 (diseases of the female reproductive system), based on the exact diagnosis (Table 2). The first discrimination of cases within an MDC is the surgical partitioning, i.e., a categorization based on whether a surgical procedure is required [23]. The immediate lower level in the CBP system comprises the DRGs. DRGs are smaller categories that categorize patient cases based on their exact diagnosis [2].

**Figure 2:**
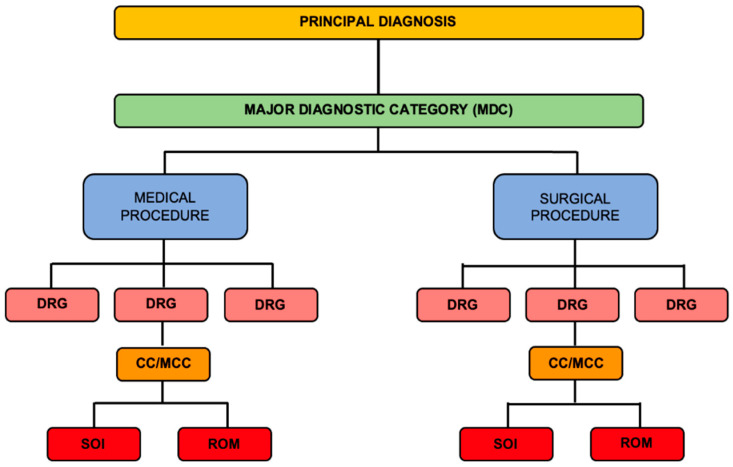
The hierarchy of the CBP model.

The number of DRGs within each of the MDCs is not fixed but depends on the complexity and the range of diseases associated with the specific body system. Consideration for the further separation of a DRG is also given to the financial differentiation of the cases, as there might be differences between treatments’ costs. In the US Medicare system, MDC 13 includes 22 different DRGs, which cover a range of procedures, from the routine ones (e.g., hysterectomy) to the most complex ones (e.g., advanced gynecologic cancers). Severity levels or (major) complications or comorbidity levels (CC/MCCs) constitute the lowest rank within the DRGs [24]. CC/MCCs further adjust the DRG on the basis of complications and comorbidities, to precisely categorize the case [19] and fairly reimburse the healthcare providers (Table 3). Lastly, a DRG is adjusted using patient-specific indexes, i.e., severity of illness (SOI) and risk of mortality (ROM) [8]. The hierarchy of the CBP system is summarized in Figure 2. The desire of the policymakers for a fair healthcare reimbursement system is also depicted through the creation of DRG outlier payments, which will be discussed later in this systematic review.

As far as gynecological cancers are concerned, ovarian cancer surgery, for example, is categorized within MDC 13, since it relates to surgery in the female reproductive system. In the DRG classification, it is further grouped, depending on the stage of the disease, the procedure required, etc., under DRG 734 “Pelvic Evisceration, Radical Hysterectomy, and Other Radical GYN Procedures with CC/MCC, including potential complications and major complications”, or under DRG 735 “Pelvic Evisceration, Radical Hysterectomy, and Other Radical GYN Procedures with CC/MCC, excluding complications”. The above differentiation depicts all levels of the CBP system, as well as the intentions of fairness in reimbursement. Another gynecological oncology example is cervical cancer treatment. This case is also categorized under MDC 13. However, the assigned DRG depends on the treatment method, specifically whether it includes surgery, radiation therapy, or chemotherapy. Therefore, the relevant DRGs include DRGs 734 and 735, similarly to ovarian cancer surgery, or DRGs 738 and 739, “Chemotherapy and radiation therapy for gynecological cancers with or without complications” [23].

### 3.3. Advantages of DRGs in the Context of Gynecologic Oncology Clinics

Assuming that participation in the DRGF scheme is voluntary and that it is not imposed through government intervention, the question as to why a healthcare provider would choose this reimbursement method seems logical. The answer to this question is multifactorial. From a financial perspective, predictability seems the most attractive incentive, especially in oncology cases, whose complexity often perplexes the financial design. Guaranteed payments for services allow clinics to develop precise budgets, while streamlining the billing process. Instead of billing for every service independently, DRGs bundle everything under a specific code and a prospective fixed payment [10]. Sloan and Steinfeld’s study proposed that economic inducement was the main determinant of the gynecologic clinics’ participation in the DRG scheme [26]. This proposition was also proved by various other studies [10]. Receiving the DRG payment, regardless of the individual treatments performed, incentivizes clinics to avoid unnecessary services and provide a cost-effective treatment. Since the additional service costs are shouldered by the providers themselves, a cost-containment approach, as well as an efficient operating policy, are adopted [7]. This efficiency is rewarding not only to the patient, who avoids a series of unnecessary tests and treatments, but also to the healthcare providers themselves, since they are permitted to keep the surplus as a profit and streamline care. Zhenyu et al. proved that DRG reform reduced the cost per admission by about 9.79% and the length of stay by about 5.35% in departments of gynecology and obstetrics [22].

The outlier payments for complex gynecological cases contribute to the attractiveness and fairness of this system. A DRGF scheme shifts the focus towards holistic outcome-centered healthcare provision. Meanwhile, DRG payment is directly linked to evidence-based national or regional guidelines, which highlight the necessary services provided in correspondence to a payment. By following these standards, care protocols are widely implemented, preventing undertreatment for lucrative purposes and standardizing high-quality care protocols [18]. Finally, DRGs help healthcare providers to align themselves with the trend towards long-term viability. Having experienced unbearable healthcare costs in the 1970s, clinics would only benefit from the proposed cost-containment strategies of the DRG scheme.

In most HICs, where healthcare is heavily regulated, and especially in countries where healthcare insurance is government-funded, participation in DRG reimbursement schemes is mandatory, should healthcare providers wish to treat publicly insured patients. This might seem like a disadvantage; however, it ensures that providers have access to a significantly larger pool of patients and that equity of healthcare, not only in the field of gynecologic oncology but across fields, is guaranteed. The mandatory DRG intervention in Germany, for instance, increased the lengths of stays by approximately 20%, defending the DRG system against under-treatment accusations [17]. Moreover, the DRG system transfers a portion of the financial risk to the providers, mitigating the insurer’s risk of overspending in complex cases. Although this seemingly negatively impacts providers, the shared financial risk relieves the insurance body and, in parallel, promotes the efficient management of hospital resources. It provides, in other words, an incentive for the amelioration of healthcare policies.

This series of incentives has led gynecologic clinics worldwide to shift towards CBP systems. Following the success of the DRG scheme in USA, most Western countries attempted to adopt a similar reimbursement strategy and adapt it to their own healthcare systems. The importance of this transition was also understood by the European Commission, which funded the research of the Euro DRG project. Its aim was to evaluate DRG-based systems, based on the quality and quantity of treatment services, while taking into consideration the financial aspects of the transition. France (T2A), Austria (LKF), Germany (gDGR), Scandinavian, and Eastern countries participated in the effort, abandoning their obsolete FFS systems. Australia (AR-DRG), China, and South Korea are also worth mentioning as key proponents [17]. The adaptation period was not equally easy in all countries and in all healthcare scenarios, e.g., oncology; nevertheless, the long-term impact is positive. The most frequent improvements in this newly established financial scheme relate to the treatment of emergency cases, while many concerns are expressed relating to high-complexity treatments.

Since 2019, the Greek DRG Institute and the German DRG administrator InEK have been working on the development and implementation of a new Greek DRG (Gr-DRG) system. The Gr-DRG system is based on the modification of the German adaptation of diagnosis-related groups (gDRG). The Gr-DRG model has been implemented since late 2023 in some Greek administrative districts and, given its success, is expected to dominate the Greek reimbursement healthcare market.

### 3.4. Application of DRG Financing in Gynecologic Oncology and Its Economic Impact

Gynecologic oncology clinics have unique characteristics and therefore present unique challenges to the reimbursement system. They specialize in treating cancers, mainly ovarian, uterine, and cervical. These treatments often demand a combination of surgical procedures, radiation therapy, and chemotherapy. The variability of each case, the differences in and individualization of each therapy type, the unexpected post-operative complications, and the adverse outcomes hinder the categorization of the cases within very few specific DRGs. The inflexible, rigid classification is a serious hurdle, as far as high-risk prolonged treatments are concerned [18]. The fixed DRG payment may not be able to cover the expenses of advanced, recurring, metastatic cancers, leaving the clinic to absorb the financial loss. Studies on resource allocation in gynecologic clinics that treated both oncology and non-oncology patients concluded that disproportional resource utilization under non-redefined oncology DRGs disincentivizes participation in the financial scheme and negatively impacts the sustainability of the healthcare system [15].

Additionally, the very low frequency and high cost variability associated with gynecological oncology cases further complicate their categorization within the DRG system [15]. Rare conditions and advanced stages of gynecologic cancers—such as recurrent or metastatic disease—are encountered infrequently in clinical practice, resulting in a limited sample size for analysis. This means there are insufficient data to accurately model cost patterns, treatment variability, and patient outcomes. Such factors are compounded by individualized care protocols, which may include experimental therapies, multi-modal treatment approaches, or extended hospital stays due to complications. This unpredictability in resource utilization makes it challenging to establish equitable DRG groupings that reflect the actual cost of care. As a result, the reimbursement model often fails to account for the financial demands of treating these patients, disproportionately affecting clinics and threatening their financial sustainability.

In their effort to ensure a fair healthcare financing system, policymakers have established additional mechanisms to ensure extra payments for the high-cost, complex cases. These mechanisms, e.g., outlier payments and add-ons, however, may not always guarantee complete recompensation for the provider in extreme cases [13]. In general, one could argue that this is not a limitation of the system, because of the profit providers gain from uncomplicated cases. Nonetheless, this is not valid in oncology clinics, in which each case is predominantly accompanied by elevated complexity. In other words, they shake DRG’s main principle, that cases under the same DRG are expected to have similar clinical progression. Factors like comorbidities and age are not sufficient in redefining the payment. Over- or underestimation of the real healthcare costs are inevitable [8].

The revenues of a clinic are linearly correlated to the number of patients treated, since the whole treatment, including complications and readmissions, are usually covered under the umbrella of the same DRG. However, the presence or absence of additional reimbursement for a readmission based on the same DRG may depend on various factors, including the specific DRG, patient severity, and any applicable penalties or incentives. Linking the entire patient condition to a fixed prospective payment, regardless of the actual services provided, prompts, in the context of gynecologic oncology, towards underservicing. Shorter lengths of stays (LOAs) and fewer follow-up tests ensure exact resource allocation, so that the clinic remains lucrative. A lower quality of treatments and early discharges, however, potentially lead to additional unexpected complications and therefore readmissions, significantly elevating the overall financial burden of the clinic. Notably, Zhenyu et al. detected the significant effect of DRG reform on the readmission risk in gynecologic clinics [22]. Whether the provider chooses to lower the quality of their services or not, the revenue margin is extremely small. Upcoding an episode of care, i.e., registering the patient’s diagnosis as a more severe one, so as to fall within the spectrum of a higher-weighted DRG, is not rare [17]. The integrity of the healthcare providers is clearly challenged. Succeeding in cost efficiency without compromising care quality seems difficult. Thus, oncology clinics may be discouraged from participating in CBP schemes, limiting both their revenues and the equity of healthcare.

The DRG model, like the FFS model, presents severe limitations in gynecologic oncology and the quality/quantity of the provided services [1].

Concerns have also been expressed regarding the adaptability of oncology clinics under the DRG reimbursement scheme to newer up-to-date approaches [1]. DRG payments are calculated based on historical costs. Cutting-edge technologies, like robotic surgeries, new drugs, and individualized cancer therapies, which can efficiently treat the cancer types associated with gynecologic oncology, are significantly more expensive than older methods. Therefore, healthcare providers are reluctant to adopt these treatments in fear of surpassing the DRG budget. Innovation is seriously impacted.

Lastly, since diagnosis is the basis of the DRG system, healthcare shifts necessarily its focus towards treating rather than preventing. For gynecologic oncology, this is very relevant, as the integration of preventative strategies can remarkably limit advanced conditions. On the one hand, clinics under the DRG scheme often launch preventive pathways for high-risk gynecologic cancer patients. For instance, if precancerous lesions are spotted during a Pap smear, the woman can be preventively treated, so that future progression, which would require resource-intensive treatment, is contained. Early diagnoses, even though they impact healthcare provider expenses, would lower the complexity of the conditions and increase the long-term profit margin of the clinics. On the other hand, all these initiatives are not led by the healthcare system itself; the organization and, of course, the expenses remain the responsibility of the providers, who aim to reduce their long-term treatment costs. As far as the gynecologic oncology field is concerned, the DRG system falls short with regard to prevention [7], as it was not initially intended for this scope. Notably, preventive DRGs and add-ons have been incorporated in the healthcare systems of many countries worldwide, with the aim of promoting prevention awareness. These preventive DRG payments may guide the system towards the right direction; however, significant steps have yet to be made.

### 3.5. Alternative/Complementary Reimbursement Schemes for Gynecologic Oncology

Diagnosis-related groups (DRGs) have played a significant role in addressing the financial challenges associated with the fee-for-service (FFS) system, contributing to improved cost efficiency and helping to enhance the financial sustainability of healthcare systems. Pure FFS retrospective financing schemes facilitated the comprehensive treatment of patients, promoting overtreating and overspending; thus, they were deemed inefficient and are now considered obsolete. DRGs have dominated the healthcare reimbursement market for over 4 decades. New reimbursement systems have been proposed and are now challenging the predominant position of DRGs. Several key differences arise when it comes to gynecologic oncology clinics’ funding [5]. After careful review of all the included studies, we summarized the advantages and disadvantages of other healthcare payment models, as presented in Table 4.

The value-based payment (VBP) models, including bundled payments and pay-for-performance among others, are meant to complement or even replace the CBP models. They set quality and outcomes as the objectives of care and incentivize cost containment, coordination between providers, prevention, and overall long-term patient health.

Bundled payments are fixed payments for an entire episode of care. The key difference between these and DRGs lie in the fact that bundled payments cover all services related to a treatment, whereas DRGs focus largely on inpatient care. This holistic reimbursement scheme proposes a coordinated treatment effort across providers, while maintaining the cost-control mindset. Bundled payments encourage an outcome-centered approach, which is valuable for comprehensive oncology treatments, that usually consist of surgery, post-operative care (chemotherapy/radiation therapy), rehabilitation, and follow ups. This payment model seems more advantageous for gynecologic oncology cases; however, the fragmentation of health systems (public and private healthcare providers) prevents its potential application.

Pay-for-performance (P4P) is another VBP reimbursement model that directly ties the quality of services provided and the treatment outcomes with fixed payments through performance targets. Healthcare providers are rewarded based on meeting specific performance benchmarks. Otherwise, they are penalized. These metrics include reducing complications and readmissions, improving patient satisfaction, incorporating preventive pathways, etc. Providers are forced to maintain elevated care standards, in order to ensure funding. Meanwhile, fixed payments for each P4P level ensure cost containment. P4P maintains the cost-containment advantage of DRGs, while eliminating their principal disadvantages: the risk of undertreating and the lack of preventive pathways for long-term patient health. However, in the context of gynecologic oncology, defining metrics may be a little more challenging, due to the prolonged periods of treatment, recurring complications and complexity. Thus, the application of this model to real-life gynecologic oncology clinics seems doubtful [1].

Population-based payment models, like capitation and global payments, differ fundamentally from DRGs, which operate on a case basis. Capitation is a payment arrangement that pays providers a fixed amount for each enrolled individual in specific time intervals (monthly, semi-annually, and annually), regardless of whether the person will utilize the healthcare services or not. The amount of remuneration varies depending on the expected healthcare utilization, i.e., high-risk populations or individuals suffering from a disease correspond to more expensive payments. Capitation retains the cost-control mindset of the DRG scheme and, in parallel, promotes preventive care and long-term health, to limit future resource-intensive expenses [1]. This is very relevant for gynecologic oncology, since complete rehabilitation is strongly correlated with identifying a cancer early in its progression. This also presents a significant advantage against DRGs.

Nonetheless, in the context of oncology, the application of capitation schemes presents significant challenges due to the high variability in treatment costs, the unpredictability of care pathways, and the high frequency of high-cost interventions. Given these limitations, it is unlikely that a pure capitation model could ever be successfully applied to oncology care without significant modifications. While there are examples of capitation being applied in oncology, they are typically partial or proposed models rather than fully implemented, comprehensive capitation systems. The most comprehensive implementation of the pure capitation model is the Oncology Care First (OCF) model, proposed by the CMS. The adaptation includes capitated payments for evaluation and management services, as well as drug administration. This represents a significant shift in oncology reimbursement, aiming to promote value-based care by providing fixed payments for specific services. While not yet widely implemented, the OCF model indicates a move towards incorporating capitation in oncology payment structures [27].

Global budget is another population-based payment model, which focuses on managing financing at an institutional level. Global payment sets an annual budget to the healthcare providers, regardless of the services they are going to provide to the patient population during the set period. This scheme encourages financial predictability and cost-efficiency, since the payment is not directly linked to the services; however, should the budget be very restrictive, the problem of underservicing arises. Population-based models, especially global budgets, can also incorporate VBP values by including specific performance goals, thereby overcoming the problem of underservicing. Global budget combined with VBP models presents a particularly promising option in the evolution of healthcare reimbursement system.

**Figure 3:**
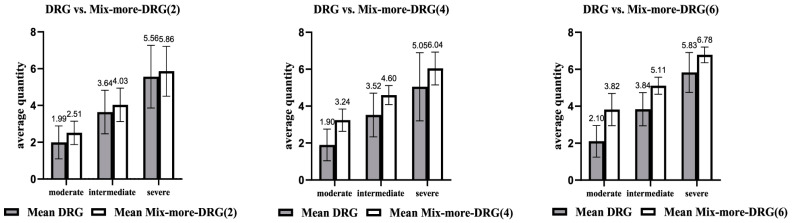
Average quantity choices in DRG and Mixed DRG payment schemes [1].

Figure 3 depicts the impact on the quantity of services from the introduction of different reimbursement schemes (VBP, population, or even retrospective (FFS)) to the existing DRG system. It compares the average quantity of healthcare services provided under DRG-based payment systems (labeled “Mean DRG”) with mixed reimbursement models (labeled “Mean Mix-more-DRG”) across different severity levels of cases: moderate, inter-mediate, and severe. Each panel represents a scenario with a different degree of integration or adjustment within the mixed DRG system, denoted as Mix-more-DRG(2), Mix-more-DRG(4), and Mix-more-DRG(6). Since the quantity of services is a direct and tangible means of measuring effective healthcare provision, it can be used to explore the efficiency of these mixed schemes. The quantity corresponding to the maximum patient benefit is defined as the optimal quantity. Taking optimal quantity as a benchmark, we can determine, based on the deviation in each case, whether the quantity of medical service provided amounts to overprovision or underprovision.

The model coexistence overall results in lower deviation from the optimal quantity of services and therefore suggests a new valid perspective. Repeating the study based on severity, since gynecologic oncology cases vary significantly in severity, gives us insights about the appropriate mixture of models in each case. In lower disease severities (and therefore lower resource consumption), prospective payments or mixed systems based on prospective payments seem more suitable. In higher disease severities, retrospective payments or mixed systems based predominantly on retrospective payments are better [1].

## 4. Discussion

DRGs have monopolized the healthcare recompensation market for over 40 years, yet insufficient data have been generated regarding the success of the DRG financing model in the context of gynecologic oncology. The inclusion of high-quality studies, according to the Newcastle–Ottawa scale, strengthens the reliability of our findings. However, the presence of moderate- and low-quality studies underscores the need for more rigorous research, particularly in evaluating DRG financing models in diverse healthcare settings. The scarce bibliographic data and the heterogenous and dissimilar study results, combined with the econometric limitations and design constraints, hinder the scientific community from drawing conclusions. The literature is predominated by descriptive non-experimental studies. Even the few that use more advanced econometric techniques are at risk of a high level of bias. A lack of unaffected controls, since the CBP system is nowadays widely implemented worldwide, low-quality pre-intervention data, and short study periods exacerbate the difficulties of reaching concrete conclusions.

The DRG system helps hospitals stay competitive and financially stable in today’s competitive healthcare environment. DRGs can be beneficial for particular types of care; this does not guarantee, however, that they are also the best approach for every healthcare scenario. The most advantageous reimbursement models depends on the perspective, on the healthcare system itself, and the goals for its population. All schemes present significant advantages, but also limitations. These benefits and challenges shift significantly when we focus on gynecologic oncology clinics. For cost containment and efficiency, DRGs work well, but at the expense of healthcare providers. For high-quality care and innovative treatment approaches, DRGs fall short in the context of complicated oncology cases. For outcome-centered care and prevention, DRGs are surely not ideal, as they mainly focus on acute episodes of care.

## 5. Conclusions

The movement from pure to mixed reimbursement models is increasingly evident, with DRGs serving as a foundational mechanism for acute inpatient care while being adapted or complemented by other models to address the complexities of oncology reimbursement. This is particularly relevant in the context of gynecologic oncology, which is characterized by its very low frequency and high cost variability, presenting unique challenges for reimbursement systems. The mixed systems provide a refined approach, balancing the strengths and weaknesses of individual payment methods. Through the tailored adjustments to the specific needs of medical departments (gynecology), disease groups (cancer), and case complexities (early/advanced stage), mixed payment models complement DRGs by addressing long-term and outpatient needs that DRGs alone may not fully support, offering a flexible and adaptive solution to current challenges of the always evolving healthcare financing landscape.

Our review emphasizes the emerging trend of integrating value-based initiatives into DRG systems, an advancement reflecting the efforts to promote holistic healthcare. However, DRGs in oncology have notable limitations, including their primary focus on acute inpatient care, which may overlook the complexities of long-term and outpatient oncology services. Value-based initiatives address these gaps by promoting comprehensive care approaches that integrate quality and outcomes across the spectrum of oncology care. These initiatives extend beyond acute episodes to encompass broader aspects of patient care, aligning financial incentives with quality outcomes. In some cases, these initiatives modify DRGs to better address inpatient care challenges, while in others they create entirely new mechanisms aimed at outpatient and longitudinal care. Within the reviewed literature, it is clear that this shift supports financial sustainability, while progressively advancing universal health coverage. In other words, in the context of gynecologic oncology, we conclude that, while DRGs have limitations in addressing the entirety of cancer care, they remain relevant and adaptable for acute inpatient services, particularly when integrated with complementary payment models.

However, several questions remain to be answered. Notably, there is limited empirical evidence on the long-term effectiveness of mixed models in complex real-world healthcare systems, particularly in low- and middle-income countries, where even DRG adoption is relatively nascent. Additionally, there is limited research on the optimal ways to integrate VBP initiatives into already existing models and how these can be adapted to countries with limited resources to avoid compromising equity or efficiency.

Future research should address these gaps by exploring the implementation challenges and outcomes associated with mixed payment systems in varying contexts. Comparative studies across healthcare systems could uncover best practices and potential pitfalls, while longitudinal analyses could assess the sustainability and impact of these models over time. Furthermore, there is a need to investigate how mixed reimbursement models influence provider behavior, patient satisfaction, and health equity, particularly in underserved populations.

By focusing on the specific context of this review, these findings highlight the crucial role of mixed reimbursement models in advancing healthcare financing strategies and of-fer a clear direction for future research to enhance and tailor these systems to diverse healthcare settings.

## Figures and Tables

**Table 1:** Characteristics of the included studies.

Author	Country	Year	Type of Study	DRG Sample	Control Group	DRG Outcome
[7]	United States	2001	NRCT	DRG patients	DRG patients	0
[12]	Germany	2011	Prospective, observational	DRG	FFS	−/+
[13]	Germany	2011	Retrospective, comparative	N/A	N/A	0/−
[14]	China	2015	CBA	Six DRG hospitals	FFS in the same hospitals	−
[15]	United States	1989	Comparative	Five DRGs	Same DRGs	−
[10]	South Korea	2010	ITS	336 DRG clinics	293 FFS clinics	+
[1]	China	2022	RCT	N/A	FFS, mixed	−
[5]	United States	2020	N/A	N/A	N/A	N/A
[16]	China	2021	Retrospective	DRG hospitals	N/A	+
[17]	Germany	2024	Retrospective, observational	DRG hospitals	Pre-DRG hospitals	+/−
[18]	United States	2020	Recommendatory	N/A	N/A	+
[19]	United States	2022	CBA	N/A	N/A	+
[20]	Germany	2010	CBA	DRG hospital	Same hospital pre-DRG	+
[8]	Belgium	2018	Retrospective	N/A	N/A	−
[21]	China	2010	CBA	One hospital	FFS model	0/−
[22]	China	2024	Retrospective	DRG hospitals	Same hospital pre-DRG	+

CBA = controlled before–after study; ITS = interrupted time series study; N/A = not applicable, RCT = randomized clinical trial; FFS = fee for service.

**Table 2:** Quality assessment using the Newcastle–Ottawa Scale.

Study	Selection (Max: 4★)	Comparability (Max: 2★)	Outcome/Exposure (Max: 3★)	Total Score (Max: 9★)	Comments
[7]	★★★★	★★	★★★	9	Strong design with DRG comparison
[12]	★★★	★★	★★	7	Prospective and observational; clear DRG vs. FFS
[13]	★★	★	★★	5	Retrospective design limits control
[14]	★★★	★	★★	6	Controlled before–after limits comparability
[15]	★★★★	★	★★	7	Comparative design; limited confounding control
[10]	★★★★	★★	★★★	9	Large DRG vs. FFS sample; robust data
[1]	★★★	★★	★★	7	Randomized trial with mixed payment model
[5]	N/A	N/A	N/A	1	Insufficient data to assess quality of the study
[16]	★★	★	★★	5	Retrospective design limits robustness
[17]	★★★	★	★★	6	Pre-DRG vs. DRG hospitals; moderate quality
[18]	★★	★	★★	5	Recommendatory; lacks strong comparability
[19]	★★★★	★	★★★	8	Controlled before–after study, high clarity
[20]	★★★★	★★	★★★	9	Strong comparative design
[8]	★★	★	★★	5	Retrospective design limits robustness
[21]	★★★	★	★	6	Single hospital limits generalizability
[22]	★★★★	★★	★★	8	Pre-DRG vs. DRG hospitals; robust data

**Table 3:** MDC 13 assignments of diagnostic codes for surgically malignant and medically malignant DRGs [25].

Uterine and Adnexal Procedures for Malignancy			
Ovarian or Adnexal Malignancy	MCC	CC	DRG
Yes	Yes		736
Yes	No	Yes	737
Yes	No	No	738
No	Yes		739
No	No	Yes	740
No	No	No	741
Malignancy, Female Reproductive System	MCC	CC	DRG
	Yes		754
	No	Yes	755
	No	No	756

**Table 4:** Advantages and challenges of other reimbursement payment models.

Payment Model	Advantages	Challenges
**Fee-for-Service**	Simplicity; Comprehensiveness.	Overtreatment; Inefficiency.
**Bundled Payments**	Care continuum; High quality of care.	Complexity; Healthcare fragmentation.
**Pay4Performance**	Efficiency; High quality of care; Prevention.	Difficulty in defining metrics.
**Capitation**	Expenditure predictability; Efficiency; Prevention.	Undertreatment.
**Global Budgets**	Expenditure predictability; Efficiency; Prevention.	Undertreatment.

## Data Availability

This study did not create or analyze new data, and data sharing does not apply to this article.
